# Competence of *Cimex lectularius* Bed Bugs for the Transmission of *Bartonella quintana*, the Agent of Trench Fever

**DOI:** 10.1371/journal.pntd.0003789

**Published:** 2015-05-22

**Authors:** Hamza Leulmi, Idir Bitam, Jean Michel Berenger, Hubert Lepidi, Jean Marc Rolain, Lionel Almeras, Didier Raoult, Philippe Parola

**Affiliations:** 1 Aix Marseille Université, Unité de Recherche en Maladies Infectieuses et Tropicales Emergentes (URMITE), UM63, CNRS 7278, IRD 198 (Dakar), Inserm 1095, World Health Organization (WHO) Collaborative Center for Rickettsioses and Other Arthropod-Borne Bacterial Diseases, Marseille, France; 2 Ecole Nationale Supérieure Vétérinaire d’Alger, Alger, Algérie; 3 Université de Boumerdes, Laboratoire VALCORE, Faculté des Sciences, Boumerdes, Algérie; 4 Université de Bab Ezzouar, Laboratoire d’Ecologie et Environnement, Alger, Algérie; United States Army Medical Research Institute of Infectious Diseases, UNITED STATES

## Abstract

**Background:**

*Bartonella quintana*, the etiologic agent of trench fever and other human diseases, is transmitted by the feces of body lice. Recently, this bacterium has been detected in other arthropod families such as bed bugs, which begs the question of their involvement in *B*. *quintana* transmission. Although several infectious pathogens have been reported and are suggested to be transmitted by bed bugs, the evidence regarding their competence as vectors is unclear.

**Methodology/Principal Findings:**

Bed bugs at the adult and instar developmental stages were fed three successive human blood meals inoculated with *B*. *quintana* bacterium from day one (D1) to D5; subsequently they were fed with pathogen-free human blood until the end of the experiment. Bed bugs and feces were collected in time series, to evaluate their capacities to acquire, multiply and expel viable *B*. *quintana* using molecular biology, immunohistochemistry and cultures assays. *B*. *quintana* was detected molecularly in 100% of randomly selected experimentally infected bed bug specimens (D3). The monitoring of *B*. *quintana* in bed bug feces showed that the bacterium was detectable starting on the 3^rd^ day post-infection (pi) and persisted until day 18±1 pi. Although immunohistochemistry assays localized the bacteria to the gastrointestinal bed bug gut, the detection of *B*. *quintana* in the first and second instar larva stages suggested a vertical non-transovarial transmission of the bacterium.

**Conclusion:**

The present work demonstrated for the first time that bed bugs can acquire, maintain for more than 2 weeks and release viable *B*. *quintana* organisms following a stercorarial shedding. We also observed the vertical transmission of the bacterium to their progeny. Although the biological role of bed bugs in the transmission of *B*. *quintana* under natural conditions has yet to be confirmed, the present work highlights the need to reconsider monitoring of these arthropods for the transmission of human pathogens.

## Author Summary

*Bartonella quintana*, the etiologic agent of trench fever and other human diseases, is known to be transmitted by the feces of body lice. Recently, the DNA of this bacterium has been detected in bed bugs. Several pathogens have been associated and suggested to be transmitted by bed bugs, despite the insufficient evidence to support this vector role. The aim of the present study was to assess the competence of bed bugs in the transmission of *B*. *quintana* using an experimental artificial model of infection. To this end, bed bugs were fed with human infected blood meals. On the 3^rd^ day post-infection (dpi) *B*. *quintana* was detected molecularly in 100% of experimentally infected bed bug. The bacterium was also detectable in bed bug feces starting on the 3^rd^ dpi and persisted until 18±1 dpi. Although immunohistochemistry assays localized the bacteria to the gastrointestinal bed bug gut, *B*. *quintana* was also detected in the first and second instars larva. The present work highlights the need to reconsider monitoring of bed bugs for the transmission of pathogens.

## Introduction

*Bartonella quintana* is a fastidious gram-negative bacterium that is regarded as a re-emerging human pathogen [1]. *B*. *quintana* DNA has been detected in the dental pulp of a 4000-year-old man [2] and in lice found in a mass grave of Napoleon’s soldiers in Lithuania, which suggests that many of the soldiers were affected by trench fever [3]. Trench fever was the first described clinical manifestation of *B*. *quintana* infection, and it affected thousands of soldiers during World Wars I and II [3]. Subsequently, *B*. *quintana* has been identified as an agent of bacillary angiomatosis in AIDS patients [4], endocarditis [5,6], chronic bacteremia [7,8], and chronic lymphadenopathy [9]. The severity of *Bartonella* infection correlates with the immune status of the patient; the clinical manifestations can range from benign and self-limited to severe and life-threatening disease [10]. Although body lice are considered as the main vector of *B*. *quintana* [11], this bacterium has also been found in other arthropods such as head lice [12,13], ticks [14] and mites [15]. Recently, after the detection of *B*. *quintana* DNA in fleas [16], it was experimentally demonstrated that the cat flea, *Ctenocephalides felis*, could acquire and excrete viable *B*. *quintana* in their feces [17]. These results supported the likely vector role of fleas in trench fever or other clinical manifestations caused by *B*. *quintan*a [17].

The recent detection of *B*. *quintana* DNA in *Cimex hemipterus* (tropical bed bugs) collected from two prisons in Rwanda indicated that bed bugs could be involved in the transmission of *B*. *quintana* [18]. This raises the question of whether *C*. *lectularius* (common bed bug) could acquire and excrete viable *B*. *quintana* and thus constitute a potential competent vector. For this purpose, we used an experimental model infection of *C*. *lectularius* bed bugs using three different approaches: qPCR, culture and immunohistochemistry.

## Materials and Methods

### Bacterial strain

*B*. *quintana* strain Oklahoma (ATCC 49793) [17] was used to infect the blood used to feed the bed bugs. The use, culturing and all procedures involving experimental infections of *B*. *quintana* were conducted in a Biosafety Level 2 room.

### Medium and growth condition

*B*. *quintana* strain was grown as described previously [19] on 5% Columbia sheep blood agar plates (BioMerieux, Marcy l’Etoile, France) in a humidified atmosphere at 37°C supplemented with 5% carbon dioxide (CO_2_) using the pouch of atmosphere generation system CO_2_ Gen (Oxoid Ltd by Mitsubishi Gas chemical Company Inc, Japan). After 8 to 10 days of culture, the bacteria were harvested by adding four-hundred μL of phosphate buffered saline (PBS), pH 7.2 (BioMerieux, Craponne, France). Two-hundred microliters of the pure bacterial suspension were mixed with 2 mL of whole blood, and this was used as the blood meal to infect the bed bugs. The remaining 200 μL of the bacterial suspension were diluted up to 10^–10^ and cultured to estimate the number of colony-forming units (CFU) per microliter.

### Bed bugs maintenance and supply

Since 2012, bed bugs (*Cimex lectularius*) have been maintained in a laboratory insectarium by our team at the WHO collaborative center for rickettsioses and other arthropod borne bacterial diseases in Marseille, France. This colony originated from bed bugs collected at the adult and the five instar stages from an infested apartment (Aix-en-Provence, France) using a modified Dyson DC34 hand vacuum system. They were maintained in containers kept in incubator at 60% humidity and 22°C. The bed bugs were fed once a week using citrated human blood obtained from the French Blood Establishment. Ethical approval for the use of in vitro human blood was obtained from the laboratory research ethics board of Molecular Hematology, French Blood Establishment. Two mL of blood was placed in a Hemotek artificial feeder machine (Hemotek 5W1; Discovery Workshops, Accrington, UK) covered by an artificial membrane of Parafilm M (Sigma-Aldrich, Saint-Louis, Missouri, USA) that was stretched to the twice of its length and width [20]. To prevent contamination during the experimental infection model, the Hemotek feeder and the recipient’s containers of bed bugs were introduced in a clear acrylic box.

### Bed bug infections

Two separate trials were conducted using *C*. *lectularius* drawn from the same colony at the same age. Prior to initiation of the infection, the bed bugs and their feces were shown to be free from *B*. *quintana* using qPCR.

We formed 4 groups for each trial including 2 infected (1 adults and 1 larva group) and 2 control groups (1 adults and 1 larva group); each group consisted of 30 bed bugs. In the adult vials we used 10 males and 20 females, and also larval group was composed of 30 Larva 1 (L1) bed bugs.

The concentration of *B*. *quintana* in the infected suspension composed by the bacterial suspension and the blood meal was 6 x 10^8^ CFU/mL bacteria in trial 1 and 8 x 10^5^ CFU/ mL in trial 2. Each group of bed bugs was fed 3 times in 5 days (every other day) with 200 μL of the bacterial suspension mixed with 2 mL of blood meal. The control groups were fed with 2 mL of uninfected blood mixed with 200 μL of PBS. Subsequently, all bed bug groups were fed with uninfected blood every other day starting on the 3^rd^ day post-infection (dpi) until the end of the experiment.

We tested 200 μL of the infected inoculum (the infected blood suspension that the bed bugs fed on) to ensure the presence of *B*. *quintana* in the infected blood meal using qPCR. We cultured 150 μL of the inoculum and plated dilutions up to 10^–10^ to ensure the viability and to determine the concentration of *B*. *quintana* in the infected inoculum.

### Sampling strategy

At the 3^rd^ dpi, five viable bed bugs and approximately 20 mg of feces from each group (from *B*. *quintana* exposed group of adults and instars and also from the control groups) were recovered for analysis by qPCR. Feces were collected from a sheet of paper placed on the bottom of the bed bugs containers. Culture analysis of feces and two bed bugs were also performed; both tests were used to determine the acquisition and viability of *B*. *quintana* in bed bugs and in their feces. Four adult *C*. *lectularius* from the *B*. *quintana*-exposed group were immunohistochemically analyzed to determine the bacterial localization. Four bed bugs from the control group were also analyzed and served as controls. Starting on the 5^th^ dpi, we recovered two adults and feces every 48 h to monitor the excretion of *B*. *quintana* through the end of the experiment (21^st^ dpi). We screened five eggs from the container housing the infected adults by qPCR at the 3^rd^ dpi to determine if the eggs were infected. Simultaneously, we recovered ten eggs to be reared in separate vials to obtain L1 and L2 larvae. The larvae were analyzed by qPCR to determine if any *B*. *quintana* acquisition occurred.

### DNA extraction

The DNA of individual bed bugs and their feces were extracted using an automatic EZ1 robot (QIAGEN-BioRobot_ EZ1, Tokyo, Japan) according to the manufacturer’s instructions (EZ1 DNA Tissue Kit, QIAGEN, Hilden, Germany). First, we decontaminated the surface of the bed bugs by 5 min immersion in ethanol alcohol (COOPER, Paris, France), followed by three 5 minutes immersions in sterile PBS as described previously [21]. Each bed bug was incubated overnight at 56°C in 180 μL of buffer G2 and 20 μL of proteinase K for pre-lysis followed by extraction using EZ1 robot. For all samples, the final elution volume was 100 μL.

### Real time PCR amplification

Template DNA was used in the qPCR assays targeting two specific *B*. *quintana* genes that encoded 3oxoacyl-[acyl-carrier-protein] synthase (*fabF*3) and a hypothetical intracellular effector (*yopP*) [13], which are both *B*. *quintana-*specific genes. The CFX96 (Bio-Rad, France) was used to perform each real time PCR. The qPCR was considered positive when the cycle threshold (Ct) was lower than 36 [17]. The number of *B*. *quintana* in each sample was calculated based on the DNA copy numbers. A qPCR standard curve was obtained by analyzing the *fabF3* and *yopP* systems in serial dilutions of *B*. *quintana* culture, and the standard value was determined for duplicate trials [17]. The *B*. *quintana* infection density was quantified as the ratio of the log of the transformed *fabF3* and *yopP* copy numbers per individual bed bug, feces, and blood meal. The cycle thresholds (Ct) values of [12.9; 14.5; 17.8; 22.0; 25.7; 28.9; 30.9; 34.3 and 36.0] correspond, respectively, to [4 x 10^9^; 4 x 10^8^; 4 x 10^7^ 4 x 10^5^ 4 x 10^4^; 4 x 10^2^; 4 x 10^1^ and 4] CFU/mL. Regressions formula was realized as following: Y = -0.377X + 14.236 (R² = 0.996) for *fabF*3 gene and Y = -0.372X + 14.158 (R² = 0.996) for *yopP* gene.

### Culture sampling

Approximately 500 μL of homogenized feces (20 mg in 500 μL of PBS) from groups of infected and uninfected bed bugs with 5% sheep’s blood were filtered using a 0.8 μm filter (Millex Ø 33 mm, Dominique Dutscher) and were cultured on agar plates [17]. The bodies of the bed bugs were also cultured using the same method described for the culturing of feces.

### Immunohistological analysis

Immunohistochemistry was performed on 3 μm-thick, paraffin-embedded sections of formalin-fixed bed bugs using the Ventana Benchmark autostainer (Ventana Medical Systems, Inc.) [17]. Four infected bed bugs (2 from each trial) and 4 uninfected bed bugs were analyzed (2 from each trial). After deparaffinization, each tissue section was incubated with polyclonal rabbit anti-*B*. *quintana* antibody, which was diluted 1:5000 as previously described [22].

## Results

### Acquisition of *B*. *quintana* by bed bugs

In the two trials, adults and L1 bed bugs were exposed to *B*. *quintana* three times in 5 days using *B*. *quintana*-infected blood meal. On the 3^rd^ dpi, we individually analyzed five adults and five L1 *C*. *lectularius* by qPCR. The control groups (fed on blood meal with 200 μL of PBS) were negative by qPCR for the presence of the bacterium in both trials. In the *B*. *quintana*-exposed groups, we detected *B*. *quintana* in 100% (5/5) of the adult bed bugs and in 100% (5/5) of the L1 bed bugs in both trials. The quantities of *B*. *quintana* in each individual bed bug sample per trial as determined by qPCR of the *fabF3* and *yopP* genes are given in Tables 1 and 2. Bacterial quantities ranged between 5.8 x 10^7^ CFU/ mL and 4.8 x 10^2^ CFU/ mL in trial 1 and from 2.8 x 10^6^ CFU/ mL to 6 x 10 CFU/ mL in trial 2. Feces of adults and larva bed bugs were also tested by qPCR to evaluate the presence of *B*. *quintan*a and to confirm the route of way of elimination. The results indicated the presence of the bacterium in the feces in both trials with 2.8 x 10^8^ CFU/ mL in the adult feces and 5.5 x 10^7^ CFU/ mL in the L1 feces in trial 1 and 9.1 x 10^3^ CFU/ mL in the adult feces and 2.8 x 10^6^ CFU/ mL in the L1 feces in trial 2 (Table 3).

**Table 1:** Cycle threshold (Ct) and copy number of *Bartonella quintana* detected by targeting *fabF3* and *yopP* genes in individual bed bugs per group at the 3^rd^ day post-infection in trial 1.

Bed bugs	*fabF3* gene	G1		G2		G3	G4
		**Ct**	**Copy**	**Ct**	**Copy**	**Ct**	**Ct**
**Bed bug 1**		18.7	1.5 x 10^7^	22.0	8.5 x 10^5^	>36	>36
**Bed bug 2**		20.2	4.1 x 10^6^	21.3	1.6 x 10^6^	>36	>36
**Bed bug 3**		17.2	5.8 x 10^7^	21.5	1.3 x 10^6^	>36	>36
**Bed bug 4**		17.8	3.5 x 10^7^	27.8	5.7 x 10^3^	>36	>36
**Bed bug 5**		21.0	2.2 x 10^6^	30.6	4.8 x 10^2^	>36	>36
**Feces**		15.4	2.8 x 10^8^	18.2	5.5 x 10^7^	>36	>36
**Bed bugs**	***yopP* gene**	**Ct**	**Copy**	**Ct**	**Copy**	**Ct**	**Ct**
**Bed bug 1**		18.3	2.2 x 10^7^	22.4	6.9 x 10^5^	>36	>36
**Bed bug 2**		21.1	2.0 x 10^6^	22.8	4.5 x 10^5^	>36	>36
**Bed bug 3**		17.5	4.3 x 10^7^	20.2	4.0 x 10^6^	>36	>36
**Bed bug 4**		16.4	1.2 x 10^8^	27.7	6.2 x 10^3^	>36	>36
**Bed bug 5**		20.5	3.2 x 10^6^	30.5	5.2 x 10^2^	>36	>36
**Feces**		15.6	2.3 x 10^8^	18.3	2.2 x 10^7^	>36	>36

**G1:** group of infected bed bugs adult, **G2:** group of infected bed bugs instar, **G3:** group of bed bugs adults control, **G4:** group of bed bugs instar control.

The conversion of Ct to bacteria number in positive samples was realized by the regression formula following: **y = -0.377x + 14.236** (R² = 0.996) **f**or ***fabF3* gene** and **y = -0.372x + 14.158** (R² = 0.996) for ***yopP* gene,** Ct > 36: sample considered to be negative.

**Table 2:** Cycle threshold (Ct) and copy number of *Bartonella quintana* detected by targeting *fabF3* and *yopP* genes in individual bed bugs per group at the 3rd day post-infection in trial 2.

Bed bugs	*fabF3* gene	G1		G2		G3	G4
		**Ct**	**Copy**	**Ct**	**Copy**	**Ct**	**Ct**
**Bed bug 1**		21.4	1.4 x 10^6^	21.5	1.3 x 10^6^	>36	>36
**Bed bug 2**		20.7	2.8 x 10^6^	25.1	5.8 x 10^4^	>36	>36
**Bed bug 3**		27.5	7.3 x 10^3^	22.5	5.6 x 10^5^	>36	>36
**Bed bug 4**		21.6	1.2 x 10^6^	23.8	1.9 x 10^5^	>36	>36
**Bed bug 5**		27.0	1.2 x 10^4^	33.0	6.0 x10	>36	>36
**Feces**		27.3	9.1 x 10^3^	20.6	2.8 x 10^6^	>36	>36
**Bed bugs**	***yopP* gene**	**Ct**	**Copy**	**Ct**	**Copy**	**Ct**	**Ct**
**Bed bug 1**		22.5	5.8 x 10^5^	22.0	8.7 x 10^5^	>36	>36
**Bed bug 2**		20.5	3.3 x 10^6^	24.6	9.3 x 10^4^	>36	>36
**Bed bug 3**		27.5	7.3 x 10^3^	21.0	2.1 x 10^6^	>36	>36
**Bed bug 4**		21,7	1.1 x 10^6^	23.7	2 x 10^5^	>36	>36
**Bed bug 5**		26.9	1.2 x 10^4^	34.2	2.3 x 10	>36	>36
**Feces**		27.2	5.5 x 10^4^	22.2	7.4 x 10^5^	>36	>36

**G1:** group of infected bed bugs adult, **G2:** group of infected bed bugs instar, **G3:** group of bed bugs adult control, **G4:** group of bed bugs instar control.

The conversion of Ct to bacteria number in positive samples was realized by the regression formula following: **y = -0.377x + 14.236** (R² = 0.996) **f**or ***fabF3* gene** and **y = -0.372x + 14.158** (R² = 0.996) for ***yopP* gene,** Ct > 36: sample considered to be negative.

**Table 3:** Molecular, culture, and immunohistologic methods for detection and isolation of *B*. *quintana* in blood meals, bed bugs, and their feces.

Trials	Group of	Sampling	Day 3 Post-Infection (P.I.)				Day 21 P.I.
	bed bugs (n)	(Quantity)	qPCR		Culture	Immunohisto	qPCR
			Ct/Bacteria(≈)	No. positive (%)		-chemistry	(*fabF3*)
**Trial 1**	**G 1(30)**	Blood meal^‡^ (2ml)	11.4≈6 x 10^8^CFU		+		
		Bed bugs (5)	19.0	5 (100%)	+^**(¥)**^	+(*)	-
		Feces (≈30mg)	15.4		+^**(¥)**^		-
	**G 2 (30)**	Blood meal^‡^ (2ml)	11.4≈6 x 10^8^CFU		+		
		Bed bugs (5)	24.6	5 (100%)	ND	+(*)	-
		Feces (≈30mg)	18.2		ND		-
	**G 3**	Blood meal^‡^ (2ml)	-	-	-		
	**Control**	Bed bugs (5)	-	0 (0%)	-	-	-
	**(30)**	Feces (≈30mg)	-	-	-		-
	**G 4**	Blood meal^‡^ (2ml)	-	-	-		
	**Control**	Bed bugs (5)	-	0 (0%)	ND	-	-
	**(80)**	Feces (≈30mg)	-	-	ND		-
**Trial 2**	**G 1’**	Blood meal^‡^ (2ml)	22.9≈8 x 10^5^ CFU		+		
	**(30)**	Bed bugs (5)	23.6	5 (100%)	+ ^**(¥)**^	+(*)	-
		Feces (≈50mg)	27.3		+ ^**(¥)**^		-
	**G 2’ (30)**	Blood meal^‡^ (2ml)	22.9≈8 x 10^5^ CFU		+		
		Bed bugs (5)	25.0	5 (100%)	+ ^**(¥)**^	+(*)	-
		Feces (≈50mg)	20.6		+ ^**(¥)**^		-
	**G3’**	Blood meal^‡^ (2ml)	-		-		
	**Control**	Bed bugs (5)	0 (0%)	0 (0%)	-	-	-
	**(30)**	Feces (≈50mg)	-		-		-
	**G 4’**	Blood meal^‡^ (2ml)	-		-		
	**Control**	Bed bugs (5)	0 (0%)	0 (0%)	-	-	-
	**(30)**	Feces (≈50mg)	-		-		-

**Group 1:** 30 infected bed bugs adults; **Group 2:** 30 infected bed bugs instars L1; **Group Control 1:** 30 uninfected bed bugs adults; **Control 2:** 30 uninfected bed bugs instars L1

**No:** number of bed bugs; **(+)** positive **; (-)** negative

**ND:** Not Done (because we haven’t enough feces to be cultured)**; (≈)**: approximately

**qPCR:** Quantitative real-time polymerase chain reaction

^(‡)^ Infected or uninfected blood

(*) observed in gut

^(¥)^ Confirmation by qPCR

### Localization of *B*. *quintana* in the bodies of bed bugs

**Fig 1:**
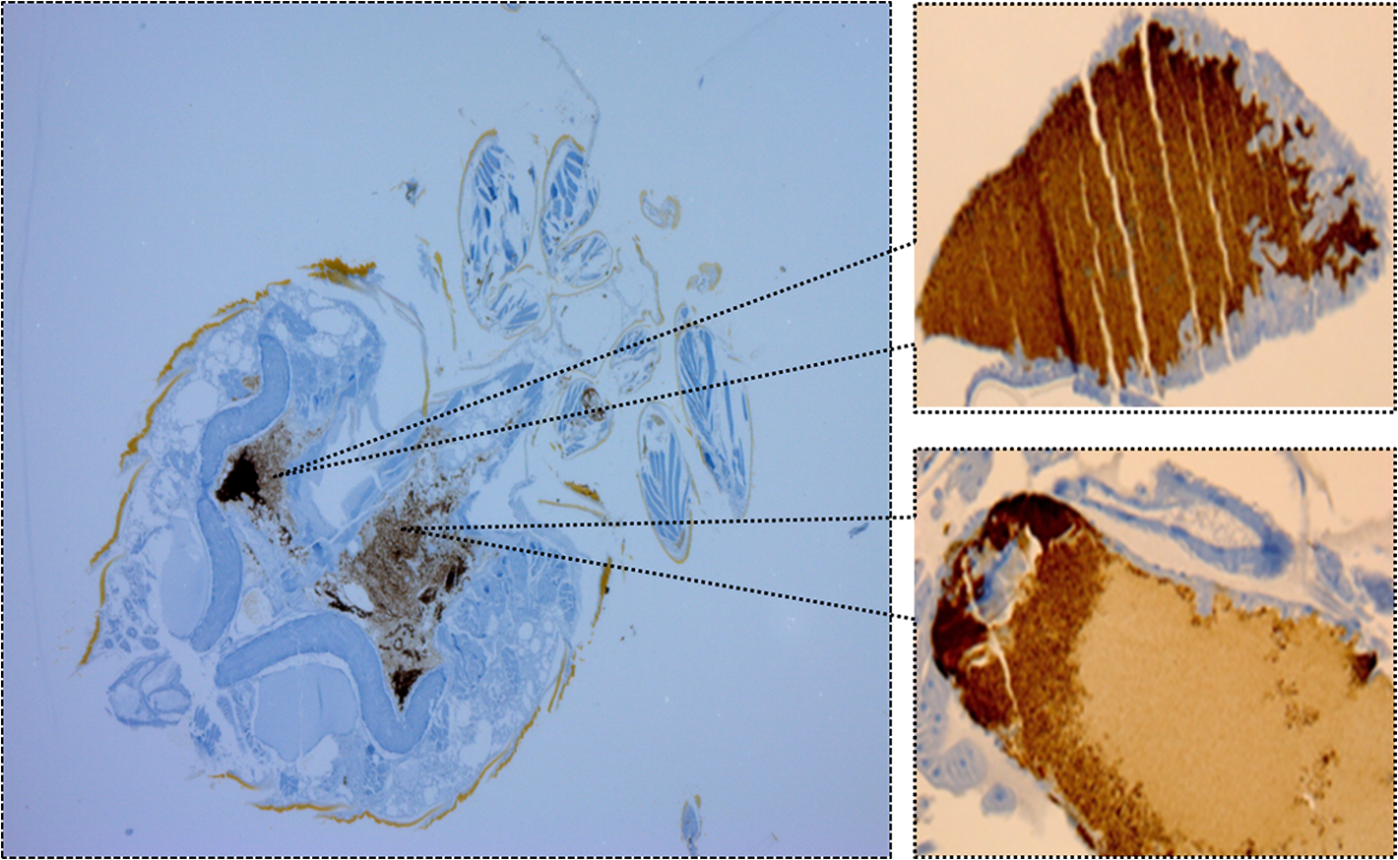
Immunohistochemistry localization of *B*. *quintana* inside the digestive tract of infected bed bugs.

Immunohistochemical analysis of the 4 tested *C*. *lectularius* (from the 3^rd^ dpi) from trial 1 and trial 2 demonstrated the presence of *B*. *quintana* as dense clusters of immunopositive microorganisms in the midgut and hindgut of the gut tract (Fig 1, Table 3).

### Evolution of *B*. *quintana* in bed bugs and their feces

#### Viability of *B*. *quintana* in bed bugs and in their feces

Cultures of homogenized and filtered feces and whole organisms from the infected adult and L1 bed bug groups were positive on the 3^rd^ dpi in both two trials. The presence of viable *B*. *quintana* was confirmed using a second culture (direct and indirect culture) and was corroborated by qPCR (Table 3).

#### Persistence of *B*. *quintana* in the body of bed bugs

**Fig 2:**
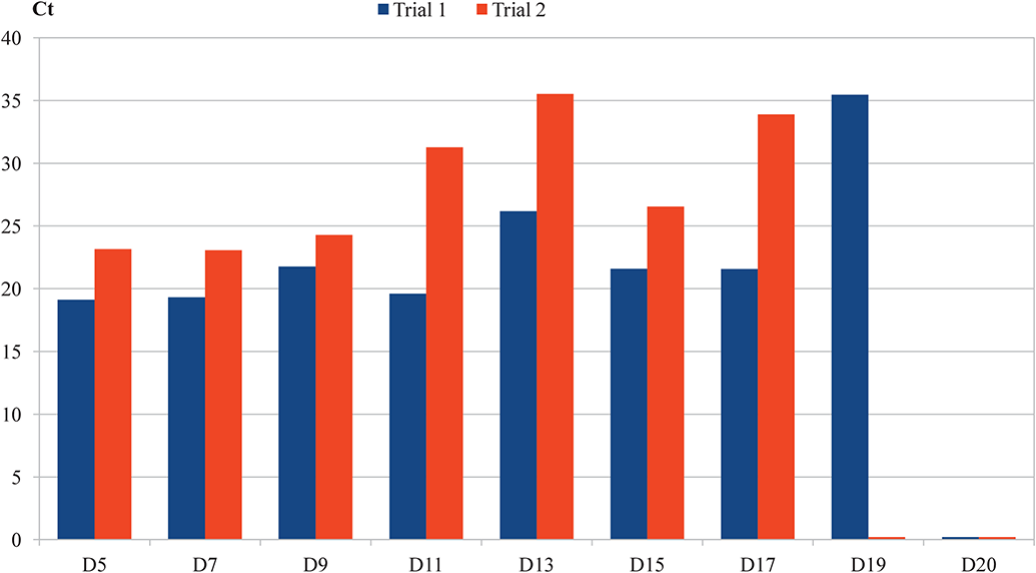
Persistence of *Bartonella quintana* in the bodies of bed bugs.

Using qPCR, we followed the presence of the bacterium in adult bed bugs from the 3^rd^ dpi until the end of the experiment. The results reported in Fig 2 demonstrate that the average number of *B*. *quintana* in the bed bugs decreased during both trials. In trial 1 (bed bugs fed with 6 x 10^8^ CFU/mL), *B*. *quintana* persist up to the 19^th^ dpi; however, in trial 2 (bed bugs fed with 8 x 10^5^ CFU/mL), *B*. *quintana* was detected until the 17^th^ dpi. We analyzed 5 eggs (recovered from the *B*. *quintana*-exposed group of adult bed bugs) at the 3^rd^ dpi and found that 2 of them were positive by qPCR (Ct [24.4, +/-2.2]). Culture analysis of the egg suspension was also positive. Ten L1 stage larvae were obtained after incubation of the eggs for 6 days; five were analyzed by qPCR and all were positive for *B*. *quintana* in both trials (the mean Ct in trial 1 was [24.4, +/-3] and [29.4, +/-1.1] in trial 2). We maintained the five remaining L1 larvae (they molt to L2 after 10 days of incubation), and one was positive in each of the trials (Ct = 21.6 in trial 1 and Ct = 31.6 in trial 2).

#### Persistence of *B*. *quintana* elimination in the feces of bed bugs

**Fig 3:**
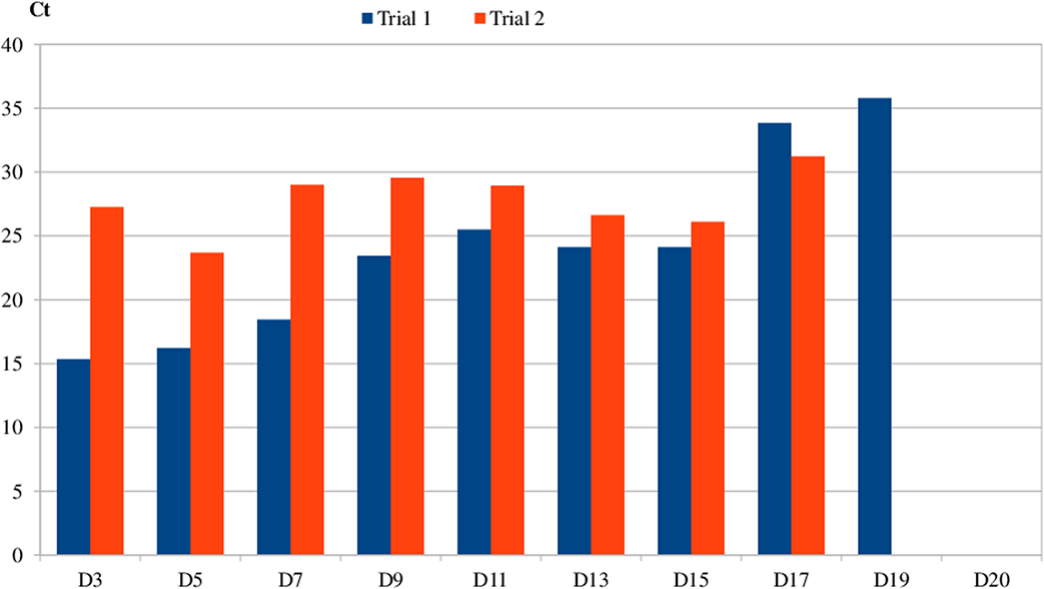
Persistence of *Bartonella quintana* in the feces of bed bug.

Using qPCR, we determined the presence of the bacterium in feces of adult bed bugs. The results reported in Fig 3 demonstrate that the average number of *B*. *quintana* bacteria in the bed bugs decreased in trial 1 up to the 19^th^ dpi and up to the 17^th^ dpi in the second trial. In addition, we noted a decreasing Ct value on the 13^th^ dpi and 15^th^ dpi compared to the 11^th^ dpi, which indicates bacterial multiplication inside the body of the bed bugs resulting in elimination at a high concentration.

## Discussion

Here, we report two experimental trials to investigate potential acquisition and transmission of *B*. *quintana* (the agent of trench fever and other diseases) by bed bugs (*C*. *lectularius*). The results show that bed bugs (adults and larva) exposed to *B*. *quintana* can acquire the bacterium and eliminate it in feces. The bed bugs maintain and shed stercorarially *B*. *quintana* for up to 17^th^ or 19^th^ dpi depending on the inoculum concentration. However, *B*. *quintana* was detected viable in feces and was shown to be alive inside the body of the bed bugs at the 3^rd^ dpi. Using immunohistochemistry, the bacterium was localized in the midgut and hindgut of the bed bugs digestive tract. Surprisingly, *B*. *quintana* was detected in eggs, L1 and L2 larvae.

In this study, we used three validated approaches. First, qPCRs was perfomed to study the acquisition and elimination of the bacterium by *C*. *lectularius*. This technique is reliable because we used a set of two qPCR systems targeting *yopP* and *fabF3*, which are known to be specific for *B*. *quintana* DNA detection, and we used negative and positive controls. Second, we cultivated the bacteria from the samples to determine if the eliminated bacteria were viable. This approach was also a validated technique [17] containing a negative and positive control. The third method was immunohistochemistry, which was used to localize the bacterium inside the body of the bed bugs. The immunohistochemistry experiments were conducted in a blinded fashion by one of us (HLi), and the results were concordant with the qPCRs results.

*Cimex lectularius* and *C*. *hemipterus* (Cimicidae: Hemipetra), commonly called bed bugs, continue to increase in scope [23,24]. In recent years, these hematophagous arthropods have undergone a major resurgence in frequency and in geographic distribution leading to clinical problems. An increasing number of infestations have been reported in Europe [25,26] [23,27] America [28], Australia [23], Asia [29,30] [31,32] and Africa [18,33].

A bite causing cutaneous lesions is the most common clinical consequence of bed bugs on public health. In addition, mental health can be affected by knowledge of a bed bug infestation in one's living environment [23]. Bed bugs are suspected of transmitting infectious agents, however there is little evidence that such transmission has ever occurred. More than 45 pathogens associated with human infection and disease have been suspected to be transmitted by bed bugs [34]. Older scientific literature cited by Goddard and de Shaso [35] suggested that bed bugs may be vectors of yellow fever, tuberculosis, relapsing fever, leprosy, filariasis [36], *kala azar* (leishmaniasis), smallpox and HIV [37,38]. *Yersinia pestis* has also noted to develop inside the body of bed bugs, *C*. *lectularius* [39,40]. Verjbitzki [40] found with animal model infection of bed bugs with high virulence strain of *Y*. *pestis* can induce death of the guinea-pigs. They found also that three bed bugs are able to convey infection [39,40]. Jordansky and Klodnitsky [41] found that the number of *Y*. *pestis* bacilli in the bed bug's stomach increased from the third to the sixth day after the infected meal [39,41]. Throughout these animal models, it may be appear that bed bugs can play an important role to convey infection of plague and perhaps other pathogens. Hepatitis B virus has also been postulated as likely candidate for possible transmission by bed bugs [42–45]. Blow et al. 2001 [45], offered evidence for stercorarial transmission of Hepatitis B viral agents from bed bugs in a time series and with transtadial transmission. Recently Salazar et al [46] assessed the vector competence of *C*. *lectularius* against *Trypanosoma cruzi* and it has been confirmed that *T*. *cruzi* was viable in bed bug feces. Goddard et al [47] have experimentally infected bed bugs with *Rickettsia parkeri* and found using immunofluorescence that the bacterium was present in the salivary gland at 15 days post infection [47]. Moreover, our laboratory recently detected *B*. *quintana* DNA in *C*. *hemipterus* collected from two prisons in Rwanda [18]. The only confirmed and known vector of *B*. *quintana* is body lice (spread through feces). However, several studies suggested that hematophagous arthropods, such as flies, lice, fleas, or ticks can acquire or transmit *Bartonella* spp. [14]. Few studies have described the kinetics of elimination and the details of transmission of these bacteria.

The results of our experiments are in agreement with many experimental infection models, such as the experimental infection of fleas with *B*. *quintana* [48], where they found that *B*. *quintana* was detected in the beginning of the 3^rd^ dpi, in fleas, as in our bed bug experimental model,. We also found that *B*. *quintana* was viable in feces and decreased gradually after the 3^rd^ dpi, which was similarly observed using the experimental cat flea *B*. *quintana* infection model [17].

Concerning the detection of *B*. *quintana* in eggs, L1 and L2 larvae, the vertical transmission of *Bartonella* species was suggested to occur, but the transmission routes were unknown [49]. Using IHC, in the four specimens we localized the bacterium to the digestive tract but not in the ovary. The presence of *B*. *quintana* in eggs, L1 and L2 larvae may be, due to vertical non-transovarial or horizontal transmission. In our context, the transmission may have occurred by external contact of the eggs, L1 and L2 larvae with the viable *B*. *quintana* released in adult’s feces which could be strongly considered as horizontal transmission. However Morick et al, demonstrate that Bartonella-positive flea feces and gut voids are proper infection sources for flea larvae and indicate that is considered as vertical non transovarial transmission [49].

In conclusion, we showed that the bed bug *C*. *lectularius* can acquire *B*. *quintana* by feeding and release viable organisms into their feces. Therefore, bed bugs may play a role as vectors of trench fever or other diseases caused by *B*. *quintana*. Knowing that stringent criteria exist in biomedical research for indicting the roles of living agents as biologically significant reservoirs and/or vectors of pathogens [50], more studies are required to better understand *B*. *quintana* persistence in both bed bugs and their feces and to understand the potential vector role of bed bugs in *B*. *quintana* other bacterial infections.
