# Correction: Competence of *Cimex lectularius* Bed Bugs for the Transmission of *Bartonella quintana*, the Agent of Trench Fever

**DOI:** 10.1371/journal.pntd.0003871

**Published:** 2015-06-15

**Authors:** Hamza Leulmi, Idir Bitam, Jean Michel Berenger, Hubert Lepidi, Jean Marc Rolain, Lionel Almeras, Didier Raoult, Philippe Parola

The following information is missing from the Funding section: This study was supported by the A*MIDEX project (n° ANR-11-IDEX-0001-02) funded by the « Investissements d’Avenir » French Government program, managed by the French National Research Agency (ANR). The funders had no role in study design, data collection and analysis, decision to publish, or preparation of the manuscript.
